# Lymphome malin non hodgkinien du sein et VIH: à propos d’un cas

**DOI:** 10.11604/pamj.2017.27.27.12279

**Published:** 2017-05-10

**Authors:** Ahmed Meklaa, Abdennasser El kharass

**Affiliations:** 1 Service de Gynécologie Obstétrique Hôpital Militaire d’Instruction Mohammed V, Rabat, Maroc; 2 Service de Radiologie, Hôpital Militaire d’Instructions Mohamed V, Rabat, Maroc

**Keywords:** Sein, lymphome, VIH, Breast, lymphoma, HIV

## Abstract

Le lymphome malin non-hodgkinien (LNH) représente 0,5% de tous les cancers du sein. Son diagnostic est essentiellement histologique. Nous rapportons un cas de LMNH du sein chez une patiente de 42 ans et chez qui la sérologie VIH est positive. L’objectif de cet article est de discuter les aspects cliniques, radiologiques et thérapeutiques de cette affection et de souligner l’importance du dépistage du VIH en cas de localisation extraganglionnaire des LMNH.

## Abstract (en)

Malignant non-Hodgkin’s lymphoma (NHL) accounts for 0.5% of all breast cancers. Diagnosis is essentially based on histology. We report the case of a 42-year old female patient with MNHL of the breast and positive HIV serology. This study aimed to highlight the clinical, radiological and therapeutic aspects of this disease and to emphasize the importance of HIV testing in patients with extraganglionar MNHL.

## Introduction

Les lymphomes non hodgkinien primitifs (LNHP) du sein sont une entité rare et représentent 2,2% de l’ensemble des lymphomes extra-ganglionnaires et moins de 0,5% des tumeurs mammaires [1]. Des facteurs de risque sont susceptibles de favoriser la survenue de cette maladie: VIH, HVC, EBV. Nous rapportons une nouvelle observation de lymphomes non hodgkinien à localisation mammaire chez une patiente avec sérologie HIV positive.

## Patient et observation

Il s'agit d'une patiente de 42 ans, divorcée, sans antécédents personnels et familiaux notables. Le début de la maladie remonte à 02 mois par l’apparition d’une tuméfaction du sein gauche augmentant progressivement de volume, associé à une tension mammaire douloureuse.

**Figure 1:**
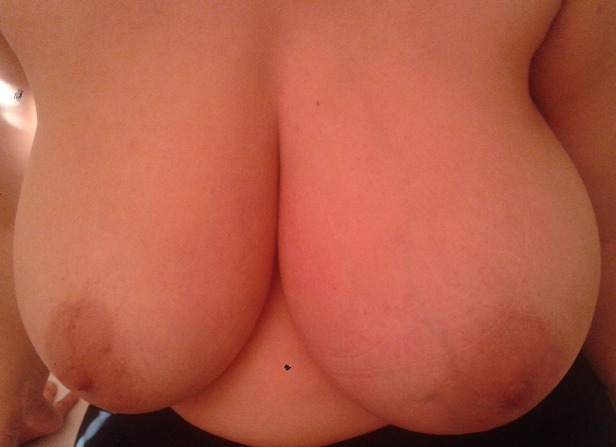
Sein gauche augmenté de volume et d’aspect inflammatoire

**A l’inspection**: sein gauche très augmenté de volume, rouge avec aspect de peau d’orange et légère rétraction mamelonnaire (Figure 1).

**À la palpation**: on trouve une masse volumineuse, dure, régulière, occupant presque tout le sein, faisant 13cm /10cm associée à des adénopathies axillaires homolatérales. Le sein controlatéral est indemne, l’examen des autres aires ganglionnaires trouve des adénopathies inguinales bilatérales; le reste de l'examen somatique est normal.

**Figure 2:**
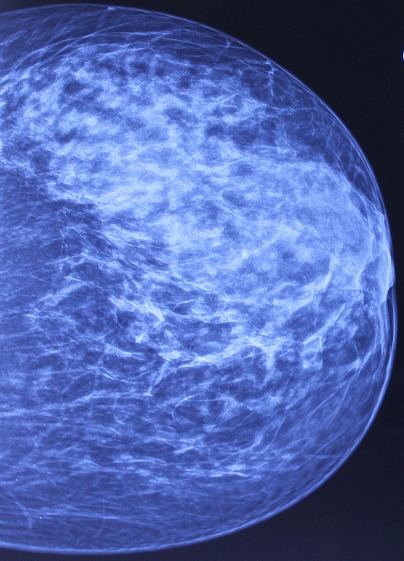
Cliché de profil montrant une opacité retromamelonnaire du sein gauche avec désorganisation architecturale

**A la mammographie**: Sein droit peu dense de type 1, Sein gauche globalement dense de type III, présentant un surcroît d’opacités rétromamelonnaire mal limite avec désorganisation architecturale associée a un épaississement sous cutanée et une rétraction mamelonnaire débutante sans microcacification sur les différentes incidences (Figure 2).

**Figure 3:**
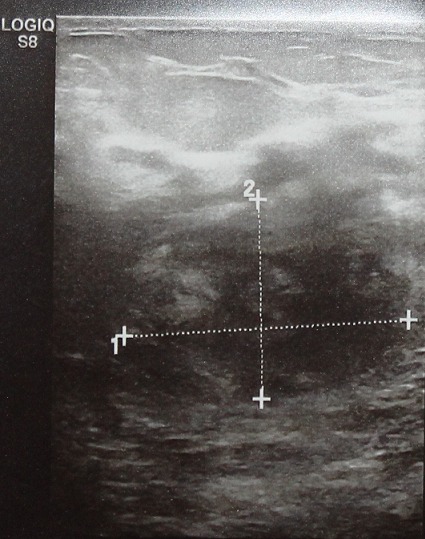
Procecuss lésionnel, hypoéchogène et hétèrogene du sein gauche

**A l’échographie**: Sein droit sans anomalie, présence d’un processus lésionnel rétromamelonnaire hypoéchogène, hétérogène hyper-vascularisée au doppler associée à de nombreuses adénopathies axillaires gauches avec épaississement de la peau et du tissu sous cutané (Figure 3).

**Figure 4:**
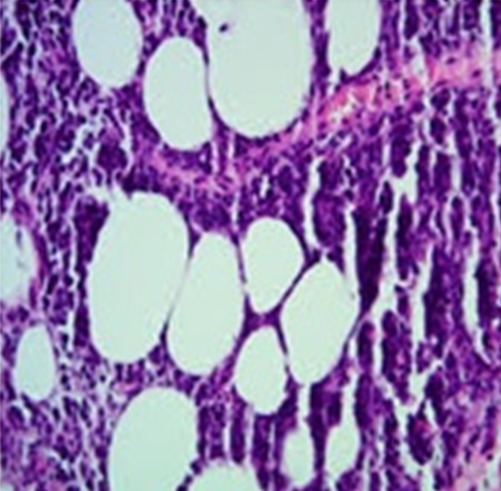
Aspect histologique d’un lymphome malin non hodgkinien à grande cellule B

L'examen histologique d'une biopsie au tricut du sein gauche objective un processus tumoral malin fait de cellules de grandes tailles pourvus d'un cytoplasme peu abondant et mal délimité. Les noyaux sont arrondis ou ovalaires, hyperchromatiques et souvent nucléolés (Figure 4). Il existe quelques mitoses anormales. L’immunomarquage de Ces cellules est négative pour l'anticorps anti-cytokératine (AE1-AE3, DAKO) et pour l'anticorps anti-CD 3 il est franchement positif pour l'anticorps anti-CD20. Le tout évoque un lymphome malin à grandes cellules B. Un bilan d’extension comportant une TDM thoraco-abdominale, une échographie abdominale et une biopsie ostéo-médullaire s’est révélé négatif. Le test du sida est revenu positif. La patiente est adressée au service d’oncologie pour prise en charge.

## Discussion

Les LMNH du sein sont très rares et représentes 0,5% de l’ensemble des tumeurs mammaires [1]. Cette néoplasie touche généralement la femme; cependant, des cas chez l’homme ont été rapportés [2]. Les facteurs de risque du lymphome malin non Hodgkinien (LMNH) sont encore mal connus, mais de multiples facteurs sont incriminés: certains virus (VIH, Epstein-Barr, hépatite C…), l’immunodépression, des maladies auto-immunes et certaines expositions environnementales comme la dioxine [3]. Sur le plan clinique Il s’agit le plus souvent d’un nodule unique, volumineux, bien limité sans phénomène inflammatoire associé [4, 5]. Plus rarement, il peut s’agit d’une tumeur inflammatoire du sein simulant une mastite carcinomateuse [5] (c’était le cas de notre patiente). Les adénopathies axillaires sont souvent retrouvées, lorsqu’elles intéressent des aires ganglionnaires inhabituelles telles que inguinales, elles devraient faire suspecter le diagnostic de LNH. La mammographie systématiquement réalisée devant tout nodule du sein est non spécifique [5]. Elle montre souvent une masse bien limitée de densité homogène d'allure bénigne, évoquant un kyste, un fibro-adénome ou une tumeur phyllode. L’échographie montre souvent une lésion hypo-échogène, homogène à contours nets et réguliers. Rarement un syndrome inflammatoire échographique est constaté. Seul l’examen histologique de la pièce opératoire, de préférence par la ponction biopsie écho-guidée permet d’affirmer le diagnostic de LNH [6]. Généralement, le diagnostic de LNH est facile, mais toujours est-il que certains points méritent d’être soulignés: le lymphome mammaire n’offre aucune particularité histologique du fait de sa localisation; l’étude extemporanée de la pièce opératoire comporte un risque d’erreur important [7]; la mastectomie d’emblée est à éviter. Une fois que le diagnostic est porté, Il est nécessaire de pratiquer un certain nombre d'examens biologiques y compris sérologie VIH, d'imagerie (radio, scanner, IRM), d'endoscopie et de biopsie, destinés à préciser le diagnostic, à évaluer l'extension du lymphome, le retentissement clinique et l'état général du patiente. Chez les femmes séropositives pour le virus de l’immunodéficience humaine (VIH), les différentes études réalisées n’ont pas mis en évidence d’augmentation de l’incidence du cancer du sein [8, 9]. Le cancer du sein présente cependant quelques particularités dans le cadre de la maladie VIH/sida: il apparaît pour des taux de lymphocytes CD4 inférieurs à 200 cellules par mm^3^, chez des femmes plus jeunes; il est volontiers bilatéral avec une histologie inhabituelle et est plus agressif avec une évolution métastatique précoce et un mauvais pronostic; La prise en charge est souvent la même que celle des patientes séronégatifs quelque soit la localisation lymphomateuse. Actuellement, la majorité des auteurs préconisent une chimiothérapie à base d'Endoxan^®^, Oncovin^®^ et Prednisone^®^ ou associée à une immunothérapie par anticorps anti-CD20 qui neutralisent les cellules cancéreuses [10]. Un traitement anti-VIH est systématiquement associe, on observe souvent chez les porteurs du VIH une forme de résistance à la chimiothérapie, qui est liée à la présence dans la membrane cellulaire d'une glycoprotéine (gp120), qui agit comme une pompe qui tend à faire sortir la molécule anticancéreuse hors de la cellule. Le pronostic des LMNH du sein est particulièrement mauvais. Le type histologique et le stade clinique de la maladie sont les deux principaux facteurs pronostiques [7, 11].

## Conclusion

Les lymphomes non hodgkinien du sein constituent une entité anatomoclinique rare. Les aspects radiographiques et cliniques ne sont pas spécifiques, le diagnostic n’est posé que sur l’histologie. Les lymphomes dont souffrent les patients séropositifs pour le virus du sida sont généralement de malignité élevée. L’étude de séries plus larges pourrait permettre de mieux codifier leur traitement et améliorer leur prise en charge.

## Conflits d’intérêts

Les auteurs ne déclarent aucun conflit d'intérêt.
